# Tissue Outcome Prediction in Patients with Proximal Vessel Occlusion and Mechanical Thrombectomy Using Logistic Models

**DOI:** 10.1007/s12975-023-01160-6

**Published:** 2023-05-30

**Authors:** Florian Welle, Kristin Stoll, Christina Gillmann, Jeanette Henkelmann, Gordian Prasse, Daniel P. O. Kaiser, Elias Kellner, Marco Reisert, Hans R. Schneider, Julian Klingbeil, Anika Stockert, Donald Lobsien, Karl-Titus Hoffmann, Dorothee Saur, Max Wawrzyniak

**Affiliations:** 1 Neuroimaging Laboratory, Department of Neurology, University of Leipzig Medical Center, Leipzig, Germany; 2 Signal and Image Processing Group, Institute for Informatics, University of Leipzig, Leipzig, Germany; 3 Department of Radiology, University of Leipzig Medical Center, Leipzig, Germany; 4 Department of Neuroradiology, University of Leipzig Medical Center, Leipzig, Germany; 5 Institute of Neuroradiology, University Hospital Carl Gustav Carus, Dresden, Germany; 6 Medical Physics, Department of Diagnostic and Interventional Radiology, Medical Center - University of Freiburg, Faculty of Medicine, Freiburg, Germany; 7 Institute for Diagnostic and Interventional Radiology and Neuroradiology, Helios Hospital Erfurt, Erfurt, Germany

**Keywords:** Computed tomography, Perfusion, Prediction, Stroke, Tissue outcome

## Abstract

Perfusion CT is established to aid selection of patients with proximal intracranial vessel occlusion for thrombectomy in the extended time window. Selection is mostly based on simple thresholding of perfusion parameter maps, which, however, does not exploit the full information hidden in the high-dimensional perfusion data. We implemented a multiparametric mass-univariate logistic model to predict tissue outcome based on data from 405 stroke patients with acute proximal vessel occlusion in the anterior circulation who underwent mechanical thrombectomy. Input parameters were acute multimodal CT imaging (perfusion, angiography, and non-contrast) as well as basic demographic and clinical parameters. The model was trained with the knowledge of recanalization status and final infarct localization. We found that perfusion parameter maps (CBF, CBV, and T_max_) were sufficient for tissue outcome prediction. Compared with single-parameter thresholding-based models, our logistic model had comparable volumetric accuracy, but was superior with respect to topographical accuracy (AUC of receiver operating characteristic). We also found higher spatial accuracy (Dice index) in an independent internal but not external cross-validation. Our results highlight the value of perfusion data compared with non-contrast CT, CT angiography and clinical information for tissue outcome-prediction. Multiparametric logistic prediction has high potential to outperform the single-parameter thresholding-based approach. In the future, the combination of tissue and functional outcome prediction might provide an individual biomarker for the benefit from mechanical thrombectomy in acute stroke care.

**Supplementary Information:**

The online version contains supplementary material available at 10.1007/s12975-023-01160-6.

## Introduction

The advent of mechanical thrombectomy has led to improved clinical outcomes in stroke patients with proximal anterior circulation vessel occlusion [1]. While thrombectomy is generally recommended in the first 6 h after symptom onset, patient selection in the extended time window represents a major challenge of modern stroke treatment. Computed tomography (CT) perfusion imaging is an established method for identifying eligible patients under these circumstances [2, 3]. After deconvolution and calculation of perfusion parameter maps [4], individual ischemic core and penumbra are visualized and quantified by thresholding these parameter maps. Established criteria [2, 3] are CBF_rel_ (i.e., cerebral blood flow relative to the unaffected hemisphere) < 30% for the ischemic core [5] and *T*_max_ (i.e., time-to-maximum of the flow-scaled residue function) > 6 s for the entire hypoperfused area including the ischemic penumbra [6], although different criteria are also applied in various commercially available software packages [7]. However, simple thresholding-based methods are not suited to exploit the full potential hidden in the high-dimensional perfusion data. Indeed, multiparametric machine learning methods (e.g., logistic regression and random forest classification) were shown to have the potential to outperform the simpler thresholding-based methods in predicting tissue outcome in case of successful (i.e., ischemic core) or unsuccessful (i.e., core and penumbra) recanalization [7–9]. These kinds of predictive models could therefore improve the selection of eligible patients with proximal anterior circulation vessel occlusion for mechanical thrombectomy by more accurate visualization and quantification of the tissue potentially salvageable (i.e., ischemic penumbra) as well as tissue that is no longer salvageable (i.e., ischemic core).

With the present study, we aimed to develop and evaluate a multiparametric mass-univariate generalized linear model (GLM) to predict tissue outcome as a function of recanalization status based on acute imaging parameters as well as basic clinical and demographic data. Using recanalization status as an input variable for model training enables the prediction of tissue outcome in new patients for both scenarios, successful and unsuccessful recanalization in terms of a “thrombectomy mismatch.” Our analyses used data from 405 stroke patients from two stroke centers. First, we determined the optimal set of input parameters for our predictive model in a step-down approach. Second, we thoroughly evaluated the predictive performance of our multiparametric mass-univariate logistic model in comparison to a single-parameter thresholding-based model focusing on clinically relevant metrics. We hypothesized that the machine learning approach would be superior to the thresholding-based approach.

## Material and Methods

### Ethics and Data Availability

The present study was conducted in accordance with the Helsinki declaration and was approved by the ethics committees of the University of Leipzig and Dresden. The retrospective analysis of data collected as part of routine care was covered by § 34 of **S**axonian hospital law. The corresponding author had full access to all data in the study and takes responsibility for its integrity and for the data analysis. Unfortunately, the datasets analyzed in the current study may not be made publicly available due to local data protection regulations. Data may be shared upon reasonable request based on a formal data sharing agreement.

### Patients

We studied patients with large vessel occlusion in the anterior circulation (intracranial internal carotid artery, M1 or M2 segment of the middle cerebral artery) who underwent mechanical thrombectomy with stent retrievers, direct thrombus aspiration, or the combination of both. We included all eligible patients admitted to the University of Leipzig Medical Center between 01/2016 and 12/2020 (“Leipzig training cohort”). For internal validation, we prospectively collected data from patients admitted between 01/2021 and 08/2021 (“Leipzig test cohort”). For external validation, we analyzed data from patients admitted to the University of Dresden Medical Center between 06/2020 and 08/2021 (“Dresden test cohort”). Inclusion criteria were available multimodal CT imaging prior to mechanical thrombectomy, i.e., noncontrast CT (NCCT), perfusion CT and CT angiography (CT-A) as well as follow-up CT or magnetic resonance imaging (MRI) suitable to delineate the final infarction. Exclusion criteria were previous large infarcts in the same territory, concurrent brain injury (e.g., severe intracerebral hemorrhage or recurrent cerebral infarction), no visible infarction on follow-up imaging despite a persisting clinical deficit, insufficient imaging quality, or missing clinical data.

We used raw DICOM imaging as well as basic demographic and clinical data obtained from the patients’ records. This data comprised age, sex, national institute of health stroke scale (NIHSS) sum score, time between stroke onset (or time when the patient was last seen well using 9:00 pm for the term “evening” and 8:00 am for the term “morning”) and initial multimodal CT imaging. Additionally, recanalization status was quantified post hoc by a board-certified neuroradiologist using the modified treatment in cerebral infarction (mTICI) scale based on digital subtraction angiography images obtained after the last thrombectomy attempt.

### Imaging

Detailed imaging protocols can be found in the Supplementary Material.

### Data Analysis

#### Preprocessing

First, all DICOM (Digital Imaging and Communications in Medicine) data was converted to Nifti-format [10]. CT perfusion parameter maps of cerebral blood volume (CBV), CBF, and *T*_max_ were calculated using VEOcore (VEObrain GmbH, Germany) with standard settings [11]. From CT angiography, source images rather than reconstructions were used. To allow for a mass-univariate approach, all imaging data was spatially normalized to the Montreal Neurological Institute (MNI) space using the Clinical Toolbox [12] for Statistical Parametric Mapping (SPM12, Wellcome Trust Centre for Neuroimaging, UK) for Matlab (Mathworks, USA). All images were coregistered to the initial NCCT. Subsequently, the initial NCCT was normalized and the resulting normalization parameters were applied to all other images. All normalization results underwent visual quality checks.

#### Lesion Delineation

Final lesion masks were semi-automatically delineated on the most appropriate CT or MRI scan (obtained around 3 days after stroke) using the Clusterize Toolbox [13] for SPM12. Each lesion was mapped three times by different trained medical students. After that, all lesion maps were carefully reviewed and, if necessary, manually corrected using MRIcron (https://www.nitrc.org/projects/mricron) by a trained neuroradiologist or neurologist. Voxels delineated in at least two of the three maps constituted the final lesion map serving as ground truth for the predictive models. Lesions were delineated on MRI scans in 50% of patients examined in Leipzig and in 18% of patients examined in Dresden and on CT scans in all other patients.

#### Predictive Models

We used a multiparametric mass-univariate logistic regression approach, which we implemented in Matlab to predict the final stroke lesions depending on recanalization status. The corresponding Matlab code is available on Github (https://github.com/afx1337/afxLogisticPrediction). The models were trained using data available in the acute setting prior to mechanical thrombectomy: multimodal CT imaging (NCCT, CT angiography, CBV, CBF, and T_max_) and basic demographic (sex, age) and clinical (NIHSS, time between stroke onset and acute imaging) information. All imaging data was smoothed with full width at half maximum (FWHM) of 0, 5, 9, and 13 mm to evaluate the optimal smoothing kernel. To predict individual benefits in tissue outcome due to thrombectomy (i.e., ischemic penumbra), we additionally included the parameter of successful recanalization (i.e., mTICI > 2a) as well as its interaction with all perfusion parameter maps in the models. This enabled us to predict two lesion maps for every patient: one assuming successful recanalization and another assuming no/unsuccessful recanalization. See Table 1 for an overview of all 14 predictor variables in the full model.

**Table 1:** Predictor variables in the full model

Category	Variable	Comment
	**Intercept**	
Imaging	NCCT	Imaging smoothed with FWHM of 0, 5, **9**, and 13 mm
	CT-A (source image)	
	**CBV**	
	**CBF**	
	**T**_**max**_	
Demographic	Age	
	Sex	
Clinical	NIHSS	
	TToImg	Time between symptom onset and acute imaging
	**Recanalization**	mTICI > 2a = successful
Interactions	**Recanalization × CBV**	
	**Recanalization × CBF**	
	Recanalization × T_max_	

Variables in bold were included in the final model. *NCCT* non-contrast computed tomography, *FWHM* full width at half maximum, *CT-A* computed tomography angiography, *CBV* cerebral blood volume, *CBF* cerebral blood flow, *T*_*max*_ time-to-maximum (of the flow-scaled residue function), *NIHSS* National Institute of Health Stroke Scale

We systematically reduced the number of variables in our model based on the predictive value in a step-down approach using 5-fold cross-validation in the Leipzig training cohort (see section “model selection”). The final model was then evaluated in the Leipzig and Dresden test cohorts. All analyses were restricted to voxels with observations (i.e., coverage by the perfusion parameter maps) in at least ten times the number of model parameters as well as with a lesion coverage of at least 5% of these patients (“GLM mask”). In addition, previous territorial lesions were excluded from the analysis.

To prevent from overfitting, we performed cross-validation and strictly separated training and test data (see Table 2 for training and test data used in the different analyses). After estimating beta values for the logistic models in each voxel in the training cohort, we were able to predict two infarction risk maps for every patient, one assuming successful and another assuming unsuccessful recanalization. Because our ground truth was binary, binarization of the infarction risk maps was required for some of the further evaluations (i.e., calculating absolute volumetric difference and Dice coefficient, see below). The optimal cutoff for binarization was derived from the training data by minimizing the mean absolute volume difference (in ml) between (in-sample) prediction and ground truth lesion map.

**Table 2:** Data used for cross-validation for the different analyses

Analysis	Training data	Test data	Folds
Model selection	Leipzig training cohort (*n* = 243/304)	Leipzig training cohort (*n* = 61/304)	5 folds
Model evaluation (internal validation)	Leipzig training cohort (*n* = 304)	Leipzig test cohort (*n* = 50)	1 fold
Model evaluation (external validation)	Leipzig training cohort (*n* = 304)	Dresden test cohort (*n* = 51)	1 fold

In addition to the logistic models, we implemented single-parameter thresholding-based models serving as a methodological baseline. In clinical practice, the ischemic core is usually defined by < 30% CBF_rel_ [5] and the ischemic penumbra (plus core) is defined by T_max_ > 6 s [6]. However, since these thresholds depend on the amount of spatial smoothing [5] and substantially vary between imaging studies [14], we did not use these fixed thresholds. The optimal thresholds (see Supplementary Fig. 1) were instead computed the same way (i.e., using cross-validation) as for the logistic models but based on the parameter maps (CBF_rel_ and T_max_) instead of the logistic model outputs.

The predicted lesion maps assuming successful recanalization were used as a surrogate for the ischemic core while the predictions assuming unsuccessful recanalization were used as indicator for the entire hypoperfused area including the ischemic penumbra. The mismatch of these two binary maps (i.e., “map(unsuccessful) AND NOT map(successful)”) corresponds to the ischemic penumbra in terms of a “thrombectomy mismatch.”

#### Model Selection

In the Leipzig training cohort, we systematically eliminated variables of low predictive value from the logistic models with a step-down approach. We started with the full model with 14 parameters (Table 1) and eliminated the parameter with the lowest mean (pseudo-)*R*^2^-value (derived from the *t*-values returned by Matlabs glmfit-function) across all voxels within the GLM mask and all five folds based on the predictions with intermediate (i.e., FWHM = 9 mm) smoothing. This step was repeated until no further eliminations were possible. The parameters intercept, CBF, T_max_, and recanalization were not allowed to be eliminated because these are the parameters of the thresholding-based models serving as methodological baseline [15]. Following the nature of linear models, interactions were eliminated prior to the corresponding main effects. We calculated the evaluation metrics introduced below for all models (using 5-fold cross-validation). The logistic model with the lowest mean absolute volume difference (prediction vs. ground truth lesion map) was chosen for further evaluation in the Leipzig and Dresden test cohort.

#### Evaluation

Three different evaluation metrics were used. First, absolute volumetric difference between the predicted lesion and the ground truth lesion map (in ml) was used as the primary evaluation metric because of the high clinical relevance of volumetric accuracy in stroke care [16]. Second, area under the curve (AUC) for the receiver operating characteristic (logistic model output vs. ground truth lesion map) was used as threshold-free metric to quantify the topographical accuracy of the infarction risk maps (per patient). Third, Dice coefficients [17] between predicted and ground truth lesion maps served as a threshold-dependent quantifier for spatial accuracy. These evaluation metrics were calculated for the logistic models as well as for the thresholding-based models and compared using Wilcoxon signed-rank tests. The evaluation was spatially restricted to the intersection of the individual voxels with available perfusion data and the GLM mask.

To evaluate the ability of the models to predict tissue outcome dependent on the recanalization success (i.e., the thrombectomy mismatch), we took advantage of the fact that the ischemic penumbra should overlap to a less extend with the ground truth lesion map in patients with successful than unsuccessful recanalization. For this aim, we calculated the relative amount of lesioned voxels (ground truth lesion map) within both compartments (core/penumbra) for both methods (logistic GLM and thresholding-based). These resulting tissue-to-infarct conversion rates were analyzed in a repeated measures analysis of variance (rmANOVA) with the factors compartment (core, penumbra), successful recanalization (true, false) and method (logistic GLM, thresholding-based) and all possible interactions. Since there were only *n* = 5 and *n* = 8 patients with unsuccessful recanalization in the Leipzig and Dresden test cohorts respectively, we performed the evaluation of the thrombectomy mismatch prediction in the Leipzig training cohort (53 patients with unsuccessful recanalization). Therefore, the results must be interpreted with caution, because the training data were also used for the feature selection process. We hypothesized that the factor compartment is more relevant in patients with successful than in patients with unsuccessful recanalization, i.e., we hypothesized a significant compartment x recanalization success interaction. Additionally, a significant three-way interaction would indicate a superiority in mismatch prediction for one of the two methods.

We also created renderings displaying predicted ischemic core and penumbra for all individual patients and models. We present illustrative cases in this manuscript; all other cases can be found in the Supplementary Information.

## Results

### Demographic and Clinical Data

Patient and imaging characteristics at admission and follow up of all included patients are summarized in Table 3.

**Table 3:** Demographic data

Variable	Leipzig training cohort (*n* = 304)	Leipzig test cohort (*n* = 50)	*p*-value (Leipzig test vs. Leipzig training)	Dresden test cohort (*n* = 51)	*p*-value (Dresden test vs. Leipzig training)
Admission					
Age (years)	74 ± 12	72 ± 14	0.55^1^	73 ± 15	0.81^1^
Sex (female)	172 (57%)	30 (60%)	0.76^2^	25 (49%)	0.36^2^
Premorbid mRS ≤ 2	268 (88%)	47 (94%)	0.33^2^	51 (100%)	0.005^2^
Pretreatment NIHSS score	16 ± 6	14 ± 5	0.02^1^	14 ± 7	0.06^1^
IVT	163 (54%)	20 (40%)	0.09^2^	17 (33%)	0.01^2^
onset-to-image time (minutes)	234 ± 269	283 ± 263	0.06^1^	467 ± 346	< 0.001^1^
NCCT ASPECTS	8.6 ± 1.9	8.2 ± 1.8	0.08^1^	7.3 ± 2.0	< 0.001^1^
Outcome					
Successful recanalization (mTICI 2b – 3)	251 (83%)	45 (90%)	0.22^2^	43 (84%)	0.84^2^
Time from CT imaging to recanalization < 180 min	237 (78%)	37 (74%)	0.47^2^	44 (86%)	0.20^2^
Follow-up modality (MRI)	144 (47%)	26 (52%)	0.55^2^	9 (18%)	< 0.001^2^
Recanalization-to-follow-up time (days)	4.3 ± 4.8	4.6 ± 6.5	0.47^1^	1.5 ± 2.2	< 0.001^1^
Ground truth infarct volume (ml)	104 ± 144	105 ± 133	0.43^1^	134 ± 136	0.005^1^
Death	43 (14%)	4 (8%)	0.37^2^	7 (14%)	1.00^2^
Discharge NIHSS score	5.9 ± 6.6	5.8 ± 6.1	0.53^1^	8.8 ± 6.8	0.004^1^
Discharge mRS ≤ 2	102 (34%)	20 (40%)	0.42^2^	12 (24%)	< 0.001^2^

Data are presented as mean ± SD and n (%). *P*-values refer to ^1^Wilcoxon rank-sum tests and ^2^Fisher's exact tests. *mRS* modified Rankin Scale, *NIHSS* National Institute of Health Stroke Scale, *IVT* intravenous thrombolysis, *NCCT ASPECTS* Alberta Stroke Program Early CT Score, *mTICI* modified treatment in cerebral infarction scale, *MRI* magnetic resonance imaging

#### Leipzig Training Cohort

Three hundred ninety-nine patients with large vessel occlusion in the anterior circulation received mechanical thrombectomy between 2016 and 2020, of whom 345 patients met the inclusion criteria. The following patients had to be excluded: (i) 19 patients with insufficient image quality due to extensive artifacts or contrast bolus delays, (ii) 16 patients with follow-up imaging on which the infarct could not be distinguished because of parenchymal hematoma with mass effect, re-infarcts, old lesions (in the same territory), or other cerebral diseases, and (iii) 6 patients with severe disability, but no visible infarct due to too early follow up imaging. Thus, 304 patients (aged 74 ± 12; 57% women) were included in the analysis.

#### Leipzig Test Cohort

Sixty-eight patients received mechanical thrombectomy between 01/2021 and 08/2021, of whom 60 had complete datasets. Four patients were excluded due to insufficient quality of imaging, five because infarcts could not be distinguished on follow-up imaging due to parenchymal hematoma with mass effect, re-infarcts or other cerebral diseases and one patient due to missing clinical data. Thus, 50 patients (aged 72 ± 14; 60% women) were included in the analysis.

#### Dresden Test Cohort

Between 06/2020 and 08/2021, 271 patients with large vessel occlusion in the anterior circulation received mechanical thrombectomy. Because in Dresden, perfusion CT is strictly performed in the time window > 6 h, only 70 patients met the inclusion criteria. In this dataset, the following patients had to be excluded: three patients with failure of the spatial normalization procedure, two patients with a second stroke in the same area, three patients with old lesions in the same territory and eleven patients with no visible infarction on follow-up CT scans despite persisting clinical symptoms. Thus, 51 patients (aged 72 ± 14; 60% women) were included in the analysis.

### Model Selection

**Fig. 1:**
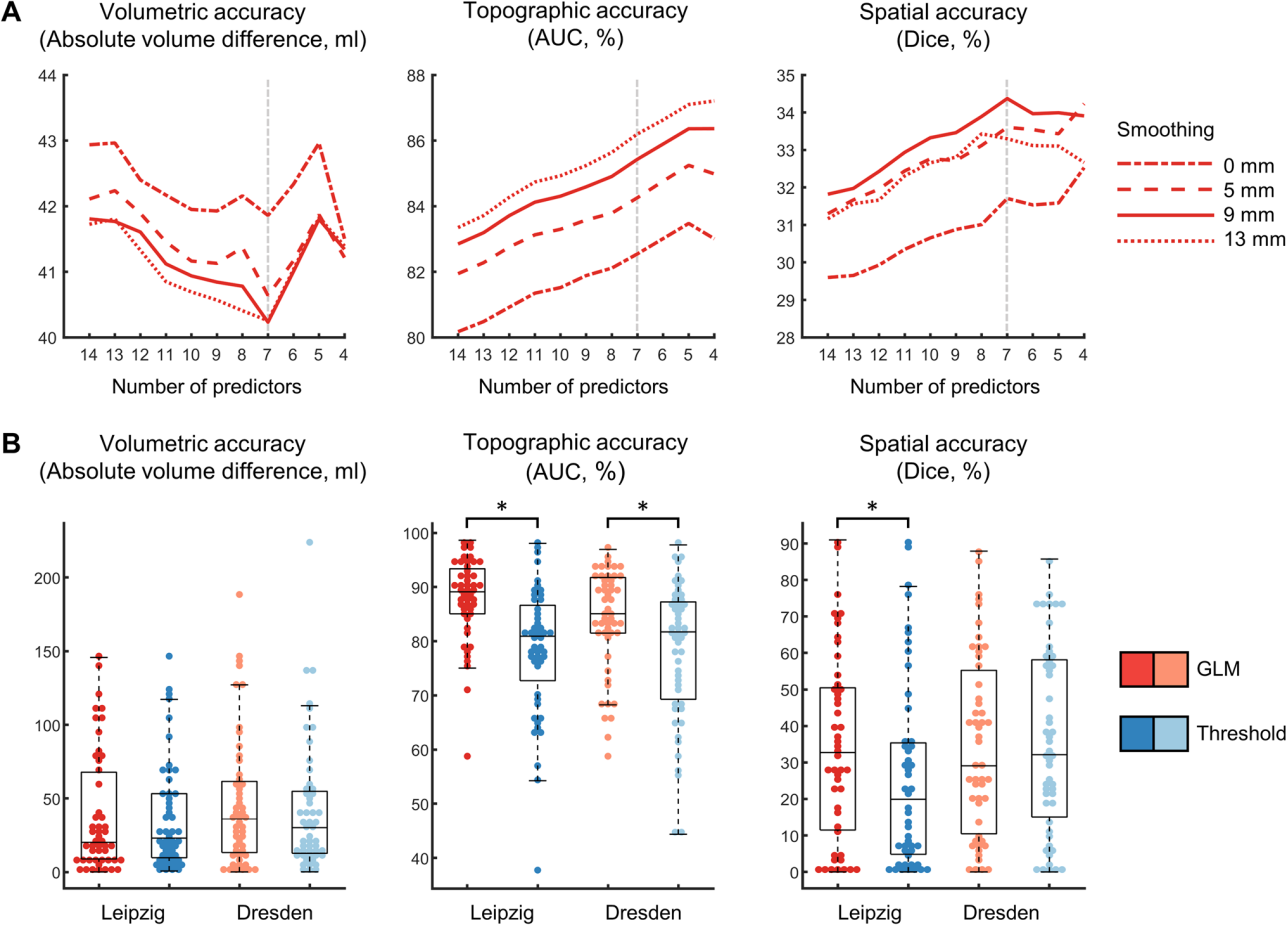
Model selection and evaluation** A** Mean evaluation metrics during model selection for the Leipzig training cohort. The gray dashed line marks the optimal number of parameters. **B** Evaluation metrics for the final logistic and thresholding-based models for the Leipzig and Dresden test cohort. AUC: area under the curve. *p < 0.001

We started with a full model including all 14 imaging (smoothed with 0, 5, 9, and 13 mm FWHM), demographic and clinical predictors (Table 1) and then searched for an optimal reduced model using a step-down approach (Fig. 1A, Supplementary Table 1). We found generally better volumetric, topographical and spatial accuracy for models based on images smoothed with 9 and 13 mm (compared to 0 and 5 mm) kernels regardless of model complexity. The best-performing logistic model in terms of volumetric accuracy was obtained after elimination of seven predictors and was based on images smoothed with 9 mm FWHM (Fig. 1A). The remaining predictors in this model were intercept, CBV, CBF, T_max_, recanalization status, recanalization status x CBV and recanalization x status CBF. The best-performing thresholding-based model was based on images smoothed with 5-mm FWHM (Supplementary Fig. 2). All further evaluations were therefore based on the optimal logistic GLM (7 parameters, FWHM = 9 mm) and on the single-parameter thresholding-based model (FWHM = 5 mm) and their comparison.

### Model Evaluation

We evaluated the final models trained with the Leipzig training cohort (*n* = 304) using independent data from the Leipzig test cohort (*n* = 50, internal cross-validation) and Dresden test cohort *(n* = 51, external cross-validation) with regard to volumetric, topographic (AUC) and spatial (Dice index) accuracy. We compared our newly developed logistic models with the thresholding-based models (Fig. 1B, Table 4). We found no differences in volumetric accuracy between the logistic and the thresholding-based models in both, the internal and the external validation (*p* = 0.78 and 0.14, Wilcoxon signed-rank test). Topographic accuracy (AUC) was significantly higher for the logistic models in both groups (*p* < 0.001, Wilcoxon signed-rank test). Spatial accuracy (Dice index) was also significantly higher in the internal validation (*p* < 0.001, Wilcoxon signed-rank test) but no difference was found in the external validation (*p* = 0.17, Wilcoxon signed-rank test).

**Table 4:** Model evaluation

Cohort	Method	Absolute volume difference (ml)	AUC (%)	Dice index (%)
Leipzig test	Logistic GLM	36.8 (26.1 – 47.4)	89.2 (87.3 – 91.1)	34.2 (26.6 – 41.8)
	Thresholding-based	35.0 (25.4 – 44.6)	80.8 (77.6 – 84.0)	27.3 (19.6 – 35.0)
		*p* = 0.78	*p* < 0.001	*p* < 0.001
Dresden test	Logistic GLM	45.0 (32.9 – 57.2)	85.5 (83.0 – 88.0)	34.3 (27.2 – 41.4)
	Thresholding-based	39.6 (28.0 – 51.2)	80.1 (76.7 – 83.5)	36.6 (29.4 – 43.8)
		*p* = 0.14	*p* < 0.001	*p* = 0.17

Mean (95%-CI), *p*-values refer to Wilcoxon signed-rank tests. *AUC* area under the curve, *GLM* generalized linear model

**Fig. 2:**
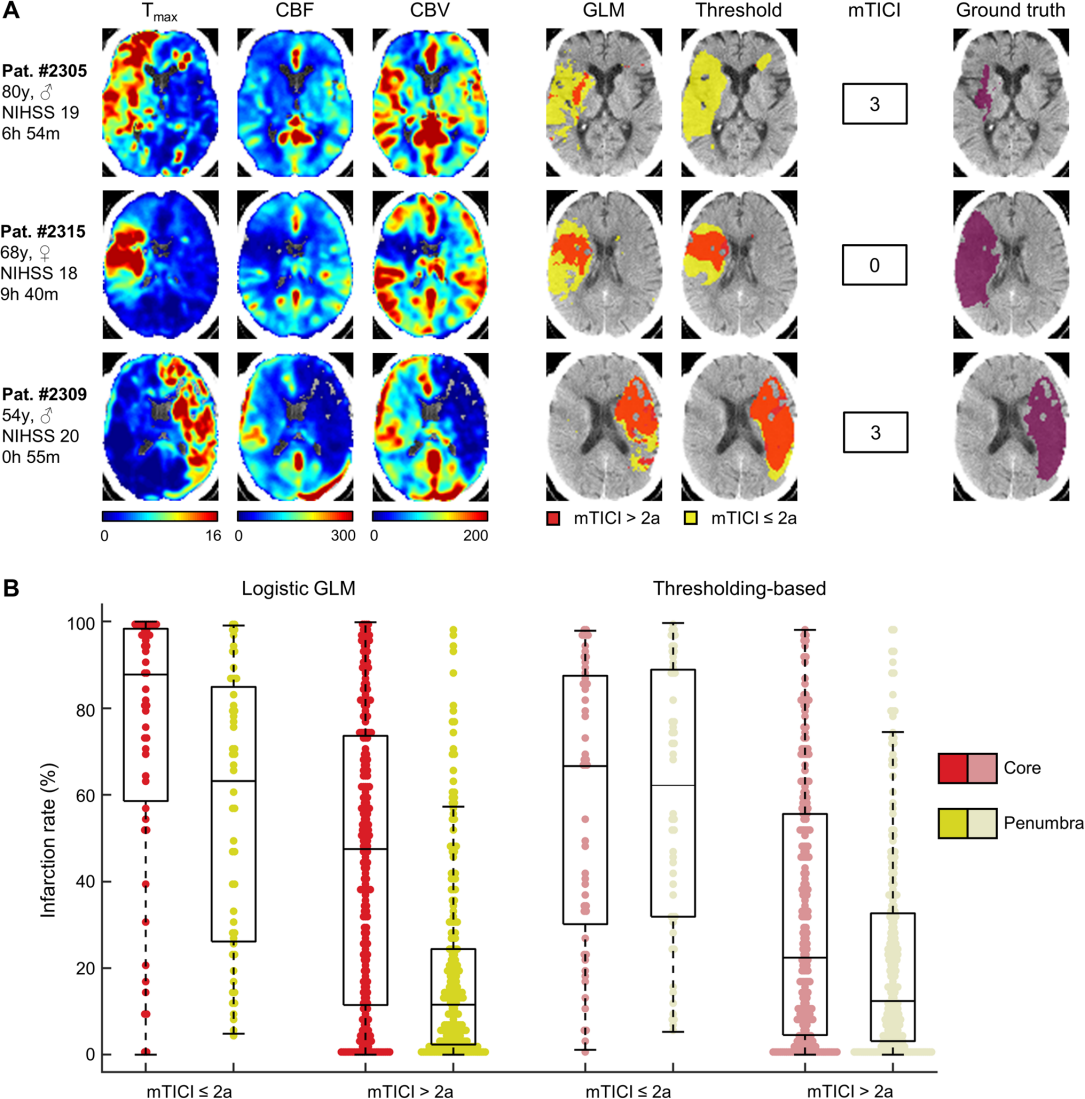
Mismatch prediction. **A** Perfusion parameter maps, logistic (GLM), and thresholding-based predictions dependent on thrombectomy success and ground truth lesion maps of three patients. Patients #2305 and #2315 represent excellent candidates for thrombectomy with large thrombectomy-mismatches while in patient #2309, marginal thrombectomy-mismatch indicates little benefit despite the good time window. Left hemisphere is displayed on the right side. **B** Tissue-to-infarct conversion rates of the different tissue compartments (core/penumbra) with regard to recanalization status and method (GLM/thresholding-based). Units: T_max_: seconds, CBF: %, CBV: %

We were also interested in the ability to predict tissue outcome dependent on the thrombectomy success for the logistic GLM and for the thresholding-based method. Prediction of this thrombectomy mismatch in three patients is illustrated in Fig. 2 A. Additionally, we calculated the amount of infarcted voxels in both compartments (Fig. 2B) and performed an rmANOVA with the factors compartment (core, penumbra), successful recanalization (true, false) and method (logistic GLM, thresholding-based). Numeric rmANOVA results are provided in Supplementary Table 2. We found a significant effect of the method: the lesions predicted by the logistic GLM were generally more likely to belong to the ground truth lesion map than those predicted by the thresholding-based method. The effect of compartment also reached significance, with regions in the ischemic core more likely to infarct than regions in the ischemic penumbra. The factor successful recanalization also explained a significant amount of variance with a higher fraction of infarcted voxels in patients with unsuccessful compared to patients with successful recanalization. The interaction method x successful recanalization, however, did not reach significance, indicating no difference in predicting recanalization effects between the two methods. In accordance with our hypothesis, the interaction compartment x successful recanalization also explained a significant amount of variance with higher effects of recanalization success in the ischemic penumbra than the ischemic core. The interaction method x compartment also reached significance, with the difference between the proportion of infarcted voxels between core and penumbra was significantly larger in the logistic GLM than in the thresholding-based method. Yet, the three-way interaction did not account for a significant amount of variance indicating that none of both methods is superior in mismatch prediction between ischemic core and penumbra.

## Discussion

We implemented and evaluated a predictive machine learning approach using mass-univariate logistic models for tissue outcome prediction depending on recanalization status. Our analyses were based on multimodal CT imaging, clinical and demographic data from 405 stroke patients with acute intracerebral proximal vessel occlusion. In the first step, we determined the optimal set of input parameters for our predictive model. In the second step, the optimal model was thoroughly evaluated using independent data.

We found that the optimal model (in terms of volumetric accuracy) included only perfusion imaging parameters and recanalization status but neither clinical or demographic variables nor NCCT and CT-A source images. In particular, no benefit was found for prediction by including the time between symptom onset and imaging or NIHSS score. Most importantly, the amount of early hypodensity on NCCT nor CT-A source images was not crucial for accurate prediction in these logistic models. This might suggest that all relevant information for predicting tissue outcome is contained in the CT perfusion imaging and that clinical and demographic variables as well as NCCT and CT-A do not contain additional information in this regard. This is of particular interest since visual scoring of early infarct signs on NCCT using Alberta Stroke Program Early CT Score (ASPECTS) still represents an important diagnostic criterion for triaging acute stroke patients in clinical routine [18]. Thus, when tissue outcome is considered a surrogate marker for functional outcome [16], patient selection for mechanical thrombectomy in the extended time window might be predominantly based on perfusion imaging findings and less on clinical parameters or NCCT.

Evaluation of the optimal model based on independent data did not implicate any differences in volumetric accuracy between the logistic and the thresholding-based models. Nevertheless, the logistic models clearly outperformed the thresholding-based models in terms of topographic accuracy (AUC). In addition, spatial accuracy (Dice) was significantly improved for the logistic model in comparison to the thresholding-based model in our internal cross-validation. This superiority of the multiparametric logistic approach is well in line with a recent study which demonstrated higher predictive performance based on precision-recall plots and mean (but not mean absolute) volume difference [7]. In our analyses, the improved spatial accuracy did not generalize to the external validation cohort. This lack of generalizability is an important limitation of our study. However, this problem likely originates from the differences in baseline characteristics between the Leipzig training cohort and the Dresden test cohort. In the Dresden test cohort, time from symptom onset to imaging was significantly longer, the ASPECTS was lower, the ground truth infarct volume larger and the early functional outcome (modified Rankin Scale as well as NIHSS) worse, although the initial clinical severity and recanalization success rate were comparable. Finally, the ground truth has been delineated earlier and more often based on CT imaging in the Dresden cohort. We therefore speculate that these patients were less represented in the training data due to local differences in performing perfusion and follow-up imaging and endovascular interventions. Additionally, the use of different scanners and protocols for obtaining perfusion CT may have been a contributing factor.

On a conceptual level, the volume of tissue potentially salvageable by intervention plays a primary role for the potential benefit to the patient (i.e., the thrombectomy mismatch). Therefore, we also compared the tissue-to-infarct conversion rates of the different compartments (ischemic core and penumbra) depending on the prediction method (logistic, thresholding-based) and recanalization success. As expected, we found significantly lower conversion rates in the penumbra compartment in patients with successful recanalization. However, this effect was not dependent on the method. Thus, despite the higher spatial accuracy of the logistic prediction approach, no difference in predicting the thrombectomy mismatch could be demonstrated with this approach.

Still, higher spatial accuracy in tissue outcome prediction using multiparametric logistic regression in comparison to thresholding-based approaches might become relevant in the future. Several previous studies [2, 3] did not take into account exact location of the predicted lesion but only considered the overall volume. However, prediction of symptoms from infarct location has improved significantly in recent years [19, 20]. Combining tissue and functional outcome prediction in the future therefore might enable predicting individual benefits in functional outcome prior to mechanical thrombectomy. Therefore, the exact location of potentially salvageable tissue might further improve the selection of patients who benefit from mechanical thrombectomy.

Finally, we would like to emphasize strengths, limitations, and some methodological considerations of our study. A main strength of our study is our large, clinically well-characterized set of real-world data from routine stroke care outside of clinical trials. The knowledge of the recanalization status of every patient is of particular value during the training of models for tissue outcome prediction. Another important strength is the focus of our model evaluation on clinically relevant metrics. We would like to highlight the difference between using volumetric difference [7] and absolute volumetric difference [21] as used in the present study. The first quantifies the tendency of the classifier to over- or underestimate the infarct volume. The latter, applied in our sturdy, quantifies actual volumetric accuracy per patient. Two predictive models can have the same mean volumetric difference but very different volumetric accuracies. Additionally, several studies in the field focus on spatial accuracy and neglect volumetric accuracy [22, 23]. We believe, that both aspects play an important role. Therefore, we optimized the models on the most relevant metric (i.e., volumetric accuracy) but also evaluated secondary metrics. This procedure, together with the cross-validation approach, rules out the possibility that the demonstrated superiority in spatial accuracy was a consequence of overfitting. Another important methodological difference compared to prior studies fitting only one single GLM to the data of all voxels [8, 23] is the mass-univariate approach which was inspired by classical statistical parametric mapping in functional neuroimaging [24]. While Kemmling and colleagues [8] used multiple maps that encoded spatial features (tissue class, territory of the middle cerebral artery), in our approach spatial information is already implicitly encoded in the models. On the other hand, the mass-univariate approach has the disadvantage of a lower number of degrees of freedom, however, this was accounted for by the large size of our training data set. To leverage the fact of similar perfusion characteristics in neighboring voxels [25], we explored different smoothing kernels and found best results with FWHM of 9 mm. Another potential methodological constraint is imbalance in the data, i.e., much more data points with no infarction in the follow-up than with infarction. Winder and colleagues [23] proposed using stratified random sampling to overcome the issue of bias in logistic models under these circumstances. Unfortunately, this approach is not feasible in combination with mass-univariate statistics. We therefore used a different approach. Instead of the theoretically motivated threshold of 0.50 [26] for binarization of our infarct risk maps, we have adjusted the thresholds for the binarization of the probabilistic output of the logistic prediction based on cross-validation (to values of around 0.38, see Supplementary Fig. 1).

Limitations of our study include uncertainties in our ground truth, mainly by mapping infarcts on subacute imaging which might have led to individual over- (mapping on MRI) or underestimation (mapping on CT and/or infarct growth after follow-up imaging) of the ground truth. Further, excluding patients with severe intracerebral hemorrhage might have biased the patient sample towards patients with better ASPECTS. Finally, our data suffered from an imbalance between fewer patients with persistent vessel occlusion compared to those with successful recanalization.

### Conclusion

Multiparametric logistic tissue outcome prediction in patients with proximal vessel occlusion in the anterior circulation primarily depends on perfusion data, but does not require clinical and demographic information or NCCT and CT-A source images. Volumetric accuracy is comparable to single-parameter thresholding-based prediction serving as methodological baseline. However, the multiparametric approach outperforms in terms of spatial (Dice) and topographic (AUC) accuracy. Spatial accuracy might be a relevant factor in future studies combining tissue and functional outcome prediction based on lesion location in terms of an individual biomarker for therapy response to mechanical thrombectomy. Finally, multiparametric generalized linear models provide the logical baseline for the evaluation of generalized nonlinear models (such as artificial neural networks). Artificial neural networks have the potential to outperform the linear models [21], but have rarely been used with consideration of recanalization status so far [27].

### Supplementary Information

Below is the link to the electronic supplementary material.

Supplementary file1 (PDF 4597 KB)
