# Supplementary material for: Tissue Outcome Prediction in Patients with Proximal Vessel Occlusion and Mechanical Thrombectomy Using Logistic Models
Source: Transl Stroke Res. 2023 May 30;15(4):739–49. doi: 10.1007/s12975-023-01160-6 (PMC11226467; doi:10.1007/s12975-023-01160-6)

Supplementary Material

Tissue outcome prediction in patients with proximal vessel occlusion and mechanical thrombectomy using logistic models

Florian Welle[1*], Kristin Stoll, MD[1*], Christina Gillmann, PhD[2], Jeanette Henkelmann, MD[3], Gordian Prasse, MD[4], Daniel P. O. Kaiser, MD[5], Elias Kellner, PhD[6], Marco Reisert, PhD[6], Hans R. Schneider, MD[1], Julian Klingbeil, MD[1], Anika Stockert, MD[1], Donald Lobsien, MD[4,7], Karl-Titus Hoffmann, MD[4], Dorothee Saur, MD[1†], Max Wawrzyniak, MD[1†]

[1]Neuroimaging Laboratory, Department of Neurology, University of Leipzig Medical Center, Leipzig, Germany
[2]Signal and Image Processing Group, Institute for Informatics, University of Leipzig, Leipzig, Germany
[3]Department of Radiology, University of Leipzig Medical Center, Leipzig, Germany
[4]Department of Neuroradiology, University of Leipzig Medical Center, Leipzig, Germany
[5]Institute of Neuroradiology, University Hospital Carl Gustav Carus, Dresden, Germany
[6]Department of Medical Physics, University of Freiburg Medical Center, Freiburg, Germany
[7]Institute for Diagnostic and Interventional Radiology and Neuroradiology, Helios Hospital Erfurt, Erfurt, Germany

[*]These authors contributed equally
[†]These authors share senior authorship

Imaging

Leipzig

CT scans were acquired at the Department of Neuroradiology, University Hospital Leipzig using a Brilliance 64-slice or Ingenuity 128-slice CT scanner (Philips Healthcare, Netherlands) with the following parameters: NCCT: slice thickness 1.25 and 5 mm, collimation 16 x 0.625 mm, 120 kV, tube current 350 mAs. Perfusion CT: 16 slices at 5 mm, collimation 64x0.325 mm, 80 kV, 100 mAs. After pre-flushing with 26 ml saline, an infusion of 50 ml radiocontrast agent (Imeron® 400) was applied followed by post-flushing with 30 ml saline each at a flow rate of 4 ml/s. With start of the contrast bolus injection, 15 volumes were acquired at 0.25 Hz. CT angiopraphy: slices number depending on anatomy, slice thickness 0.8 and 1 mm, coverage from the aortic arch to the vertex, collimation 64x0.325 mm, 100 kV, 134 mAs, bolus tracking in the aortic arch, trigger threshold 100 HU with a scan delay of 3.8 s. Protocol for contrast bolus injection is identical to CTP.

MRI scans including diffusion-weighted (DWI, voxel size 1.8x1.8x3.0 mm^3) and fluid attenuated inversion recovery (FLAIR, voxel size 0.9x0.9x3.0 mm^3) images were acquired at 3 Tesla with a Magnetom Trio Trim (Siemens, Germany).

Dresden

CT images were acquired at the Institute of Neuroradiology, University Hospital Dresden using a SOMATOM Definition Edge 128-sclice or SOMATOM Definition AS+ 128-slice CT scanner (Siemens Healthineers, Germany) with the following parameters: NCCT: slice thickness 1.5 and 6 mm, collimation 2x64x0.6 mm, 120 kV, 350 mAs. Perfusion CT: Coverage 86 mm, slice thickness 1.5/5/10 mm, collimation 32x1.2 mm, 80 kV, 200 mAs. An infusion of 35 ml radiocontrast agent (Ultravist® 370) was applied followed by post-flushing with 50 ml saline at a flow rate of 4 ml/s. With a delay of 2 s after the contrast bolus injection, 30 volumes were acquired in 46.35 s. CT angiography: slice thickness 0.6 and 1.5 mm, coverage from the aortic arch to the vertex, collimation 2x64x0.6 mm, 100 kV, 160 mAs. An infusion of 60 ml radiocontrast agent (Ultravist® 370) was applied followed by post-flushing with 30 ml saline at a flow rate of 4 ml/s. Bolus tracking in the aortic arch started with a delay of 8 s, trigger threshold 120 HU with a scan delay of 3 s.

MRI scans including diffusion-weighted (DWI, voxel size 1.0x1.0x3.0 mm^3) and fluid attenuated inversion recovery (FLAIR, voxel size 0.7x1.0x3.0 mm^3) images were acquired at 3 Tesla with a Magnetom Vida (Siemens, Germany).

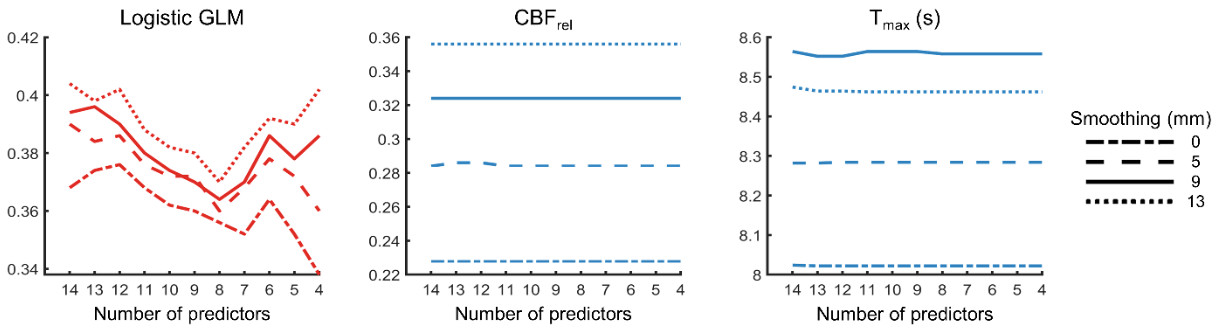

SI Figure 1. Optimal thresholds for tissue outcome prediction. The left panel shows the optimal thresholds to binarize the probabilistic output of the logistic GLM with respect to amount of smoothing and model complexity. The middle and right panel shows the optimal threshold for the single-parameter thresholding based prediction. Slight variance in thresholding-based models is caused by differences in the GLM mask. Data is based on the Leipzig test cohort using 5-fold cross-validation.

**Parameters**	**14**	**13**	**12**	**11**	**10**	**9**	**8**	**7**	**6**	**5**	**4**
Intercerpt	0.136	0.144	0.145	0.147	0.149	0.152	0.154	0.157	0.174	0.180	0.200
NCCT	0.007	0.006	0.006	0.006	0.006	**0.006**					
CT-A	0.006	0.006	0.006	0.006	**0.006**						
CBF	0.012	0.015	0.015	0.015	0.015	0.015	0.015	0.016	0.023	0.022	0.029
CBV	0.009	0.009	0.009	0.009	0.009	0.009	0.008	0.009	0.008	**0.008**	
Tmax	0.008	0.020	0.020	0.020	0.020	0.021	0.022	0.024	0.024	0.026	0.028
Age	0.006	0.005	**0.005**								
Sex	0.005	**0.005**									
NIHSS	0.008	0.007	0.007	0.007	0.007	0.007	**0.007**				
TToImg	0.006	0.006	0.005	**0.005**							
Recanalization	0.006	0.007	0.007	0.007	0.007	0.007	0.007	0.007	0.006*	0.040*	0.040
Recanalization x CBF	0.007	0.008	0.008	0.008	0.008	0.008	0.007	**0.008**			
Recanalization x CBV	0.008	0.009	0.009	0.009	0.008	0.008	0.008	0.009	**0.007**		
Recanalization x Tmax	**0.005**										
**Absolute volume difference (ml)**	41.76	41.82	41.54	41.15	40.94	40.88	40.78	40.23	40.98	41.81	41.34

SI Table 1. Parameter elimination during model selection. Mean (pseudo-)R^2 values across all voxels and folds based on images smoothed with FWHM of 9 mm. The parameter with the lowest value (bold) was eliminated in each step. *The parameter of recanalization status was not eliminated to allow for mismatch prediction.

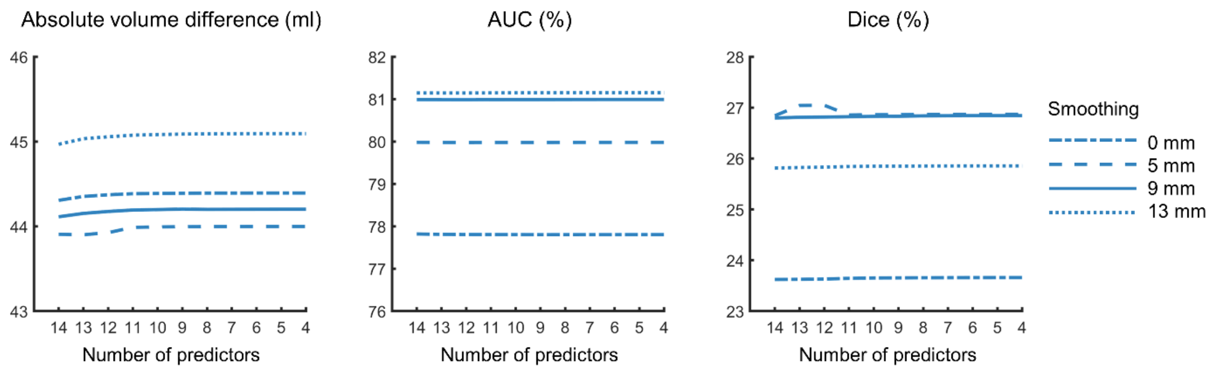

SI Figure 2. Model selection – thresholding-based. Evaluation metrics during model selection for the Leipzig training cohort. Slight variance in thresholding-based model performance across models is caused by differences in the GLM mask.

**Factor/interaction**	**F-value**	**p-value**
method	55.0	< 0.001
compartment	82.1	< 0.001
successful recanalization	66.2	< 0.001
method x successful recanalization	1.2	0.28
compartment x successful recanalization	7.1	0.01
method x compartment	89.7	< 0.001
method x compartment x successful recanalization	0.3	0.56

SI Table 2. Mismatch prediction rmANOVA results.

Individual Predictions (Leipzig test cohort)

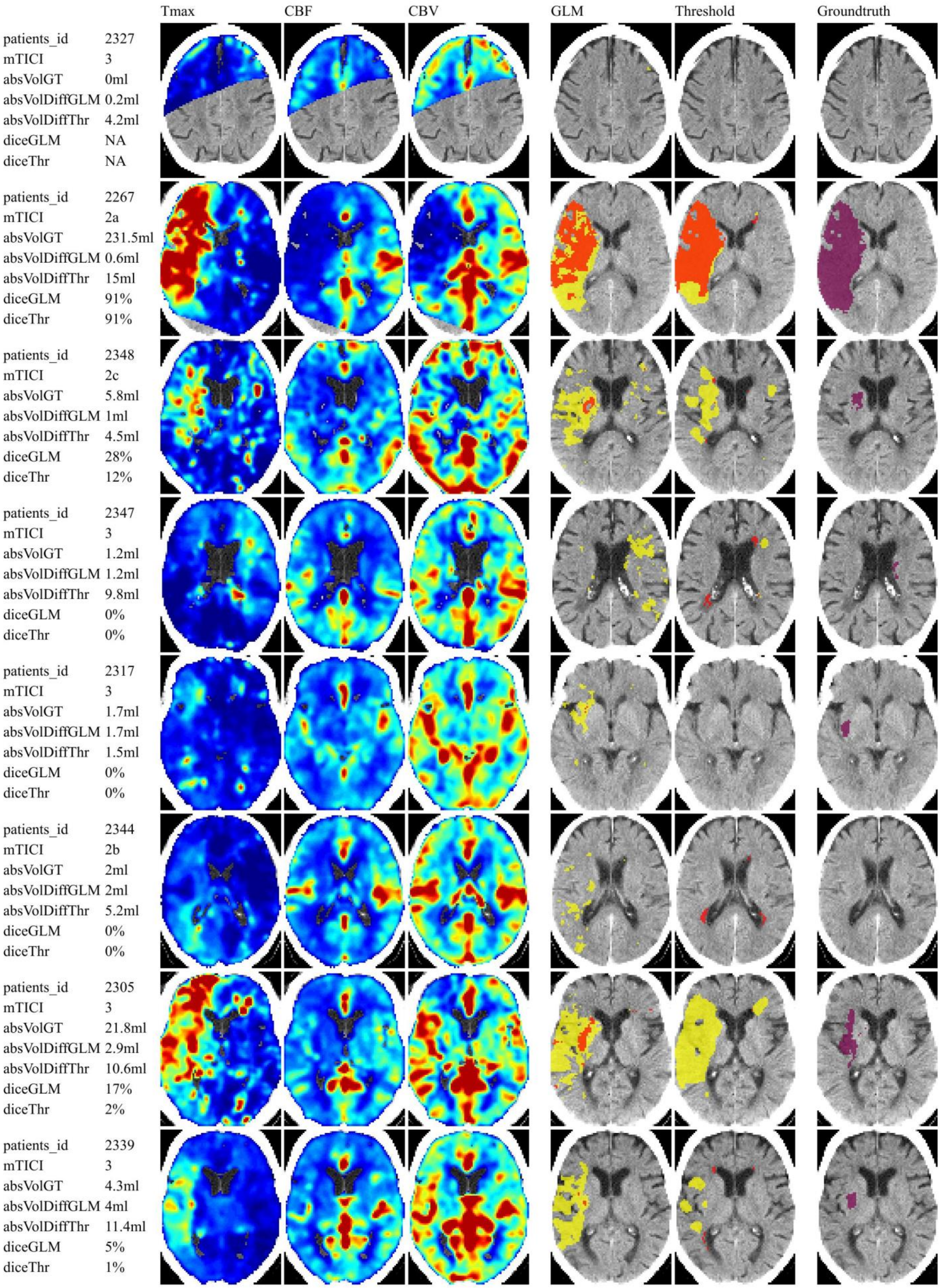

Individual Predictions (Leipzig test cohort)

patients_id	mTICI	absVolGT	absVolDiffGLM	absVolDiffThr	diceGLM	diceThr
2323	2c	30.9ml	7.7ml	20.9ml	23%	16%
2340	3	10.1ml	8.3ml	6.9ml	27%	23%
2308	3	9.3ml	8.5ml	2.4ml	12%	8%
2325	3	5.1ml	8.5ml	19.7ml	25%	2%
2349	3	5.1ml	9ml	5.8ml	28%	7%
2329	2b	35ml	9ml	16.4ml	64%	35%
2346	3	10ml	9.5ml	0.8ml	0%	29%
2326	2c	9.8ml	9.5ml	2.2ml	5%	1%

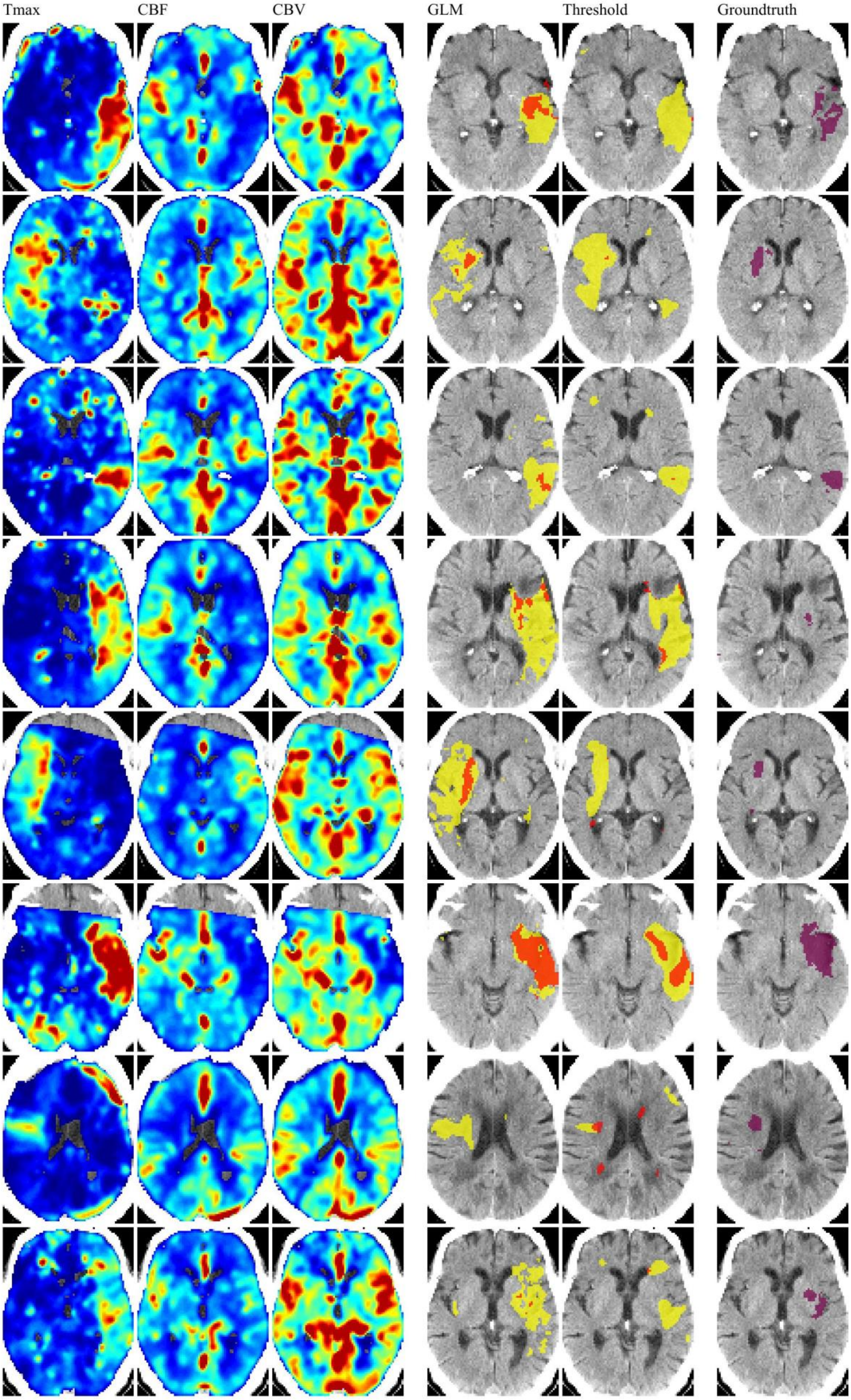

Individual Predictions (Leipzig test cohort)

patients_id	mTICI	absVolGT	absVolDiffGLM	absVolDiffThr	diceGLM	diceThr
2322	3	37.4ml	13.5ml	18.2ml	60%	33%
2320	3	15.5ml	14.5ml	11ml	3%	0%
2338	3	18.7ml	14.9ml	16.4ml	17%	2%
2312	1	58.7ml	16.8ml	53ml	43%	8%
2318	3	16.8ml	16.8ml	8.5ml	0%	6%
2441	3	9.6ml	17.2ml	5.1ml	23%	8%
2335	3	59.6ml	17.2ml	8.5ml	63%	49%
2341	2c	32.3ml	18.6ml	28.2ml	39%	6%

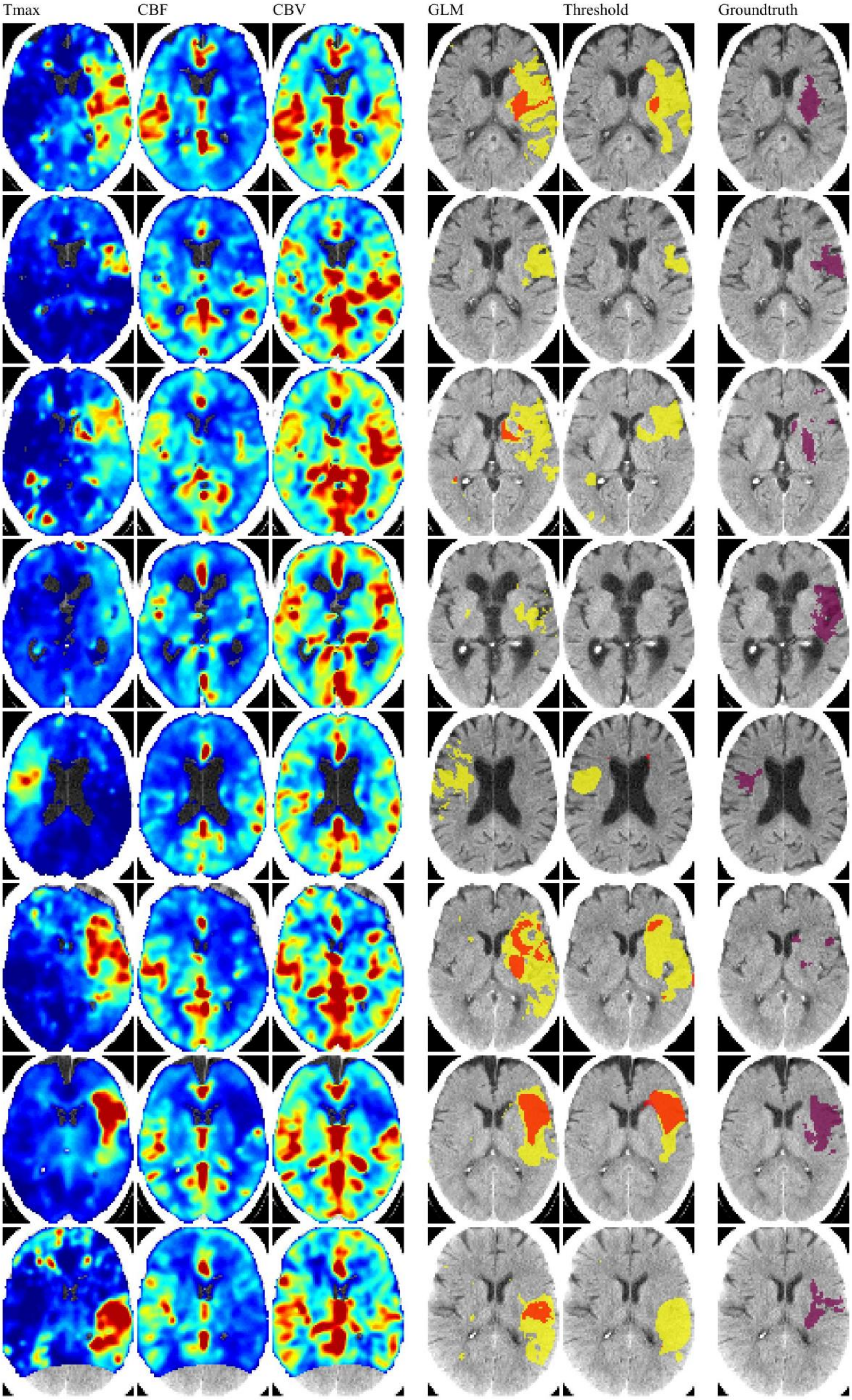

Individual Predictions (Leipzig test cohort)

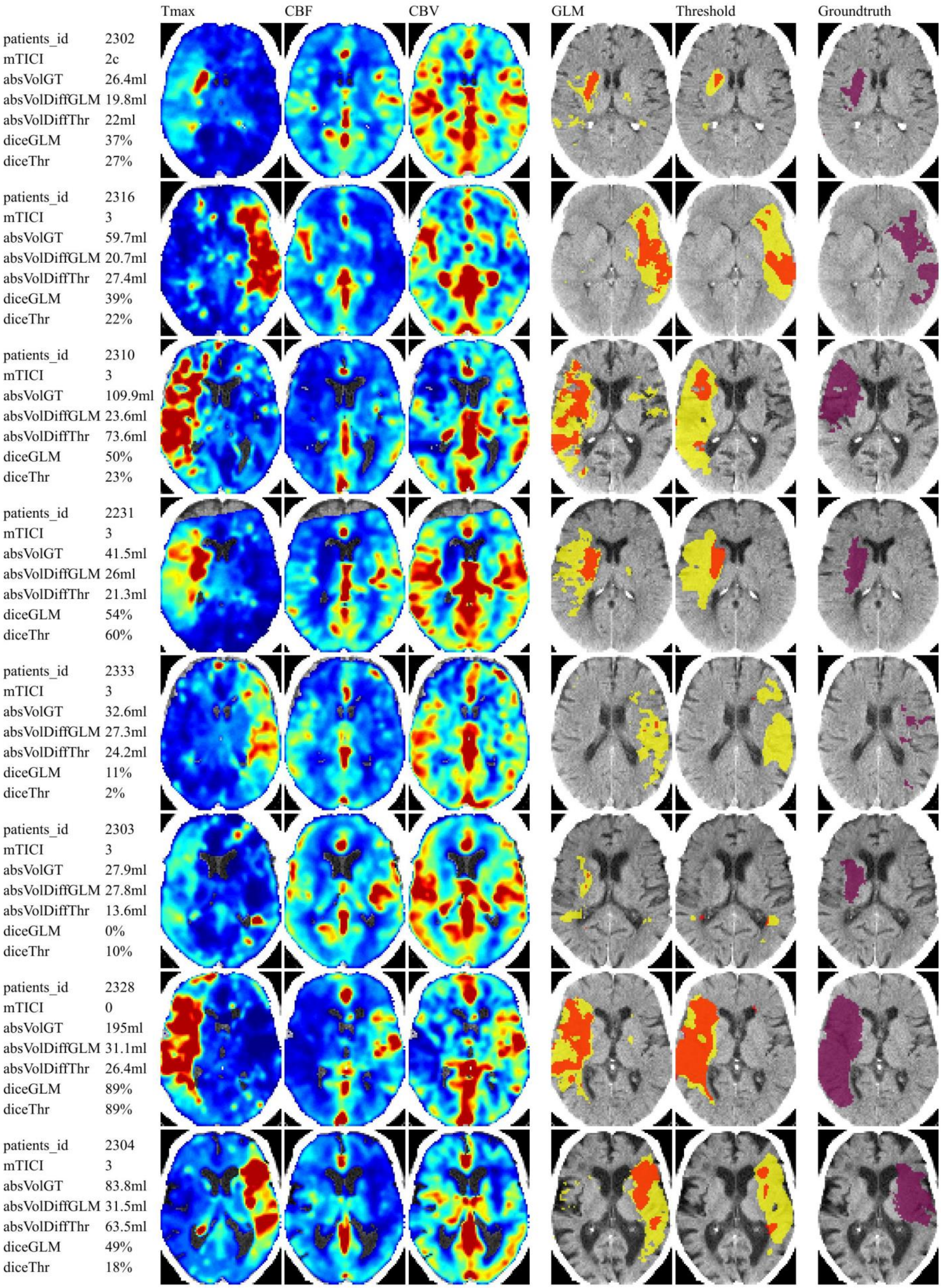

Individual Predictions (Leipzig test cohort)

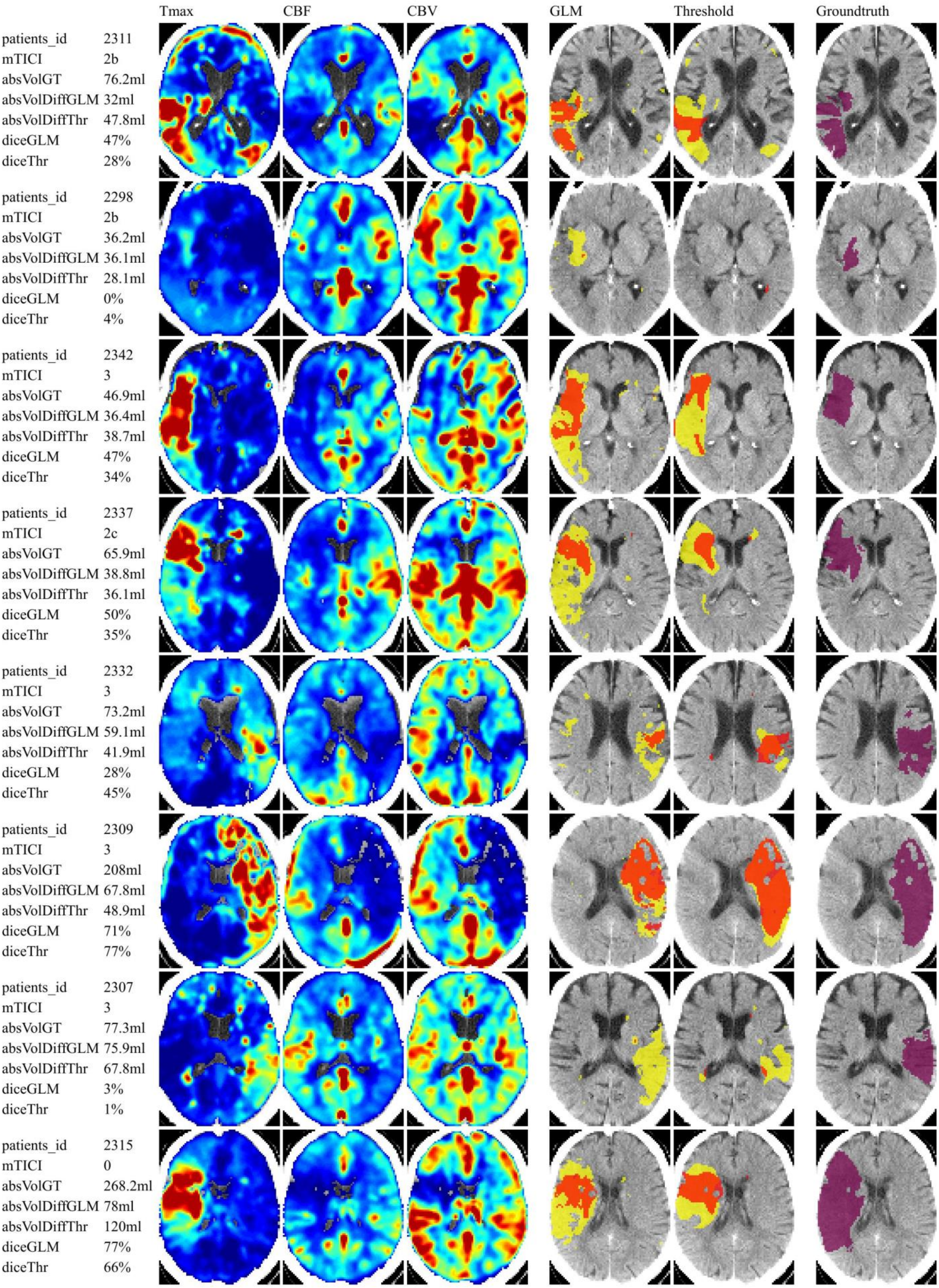

Individual Predictions (Leipzig test cohort)

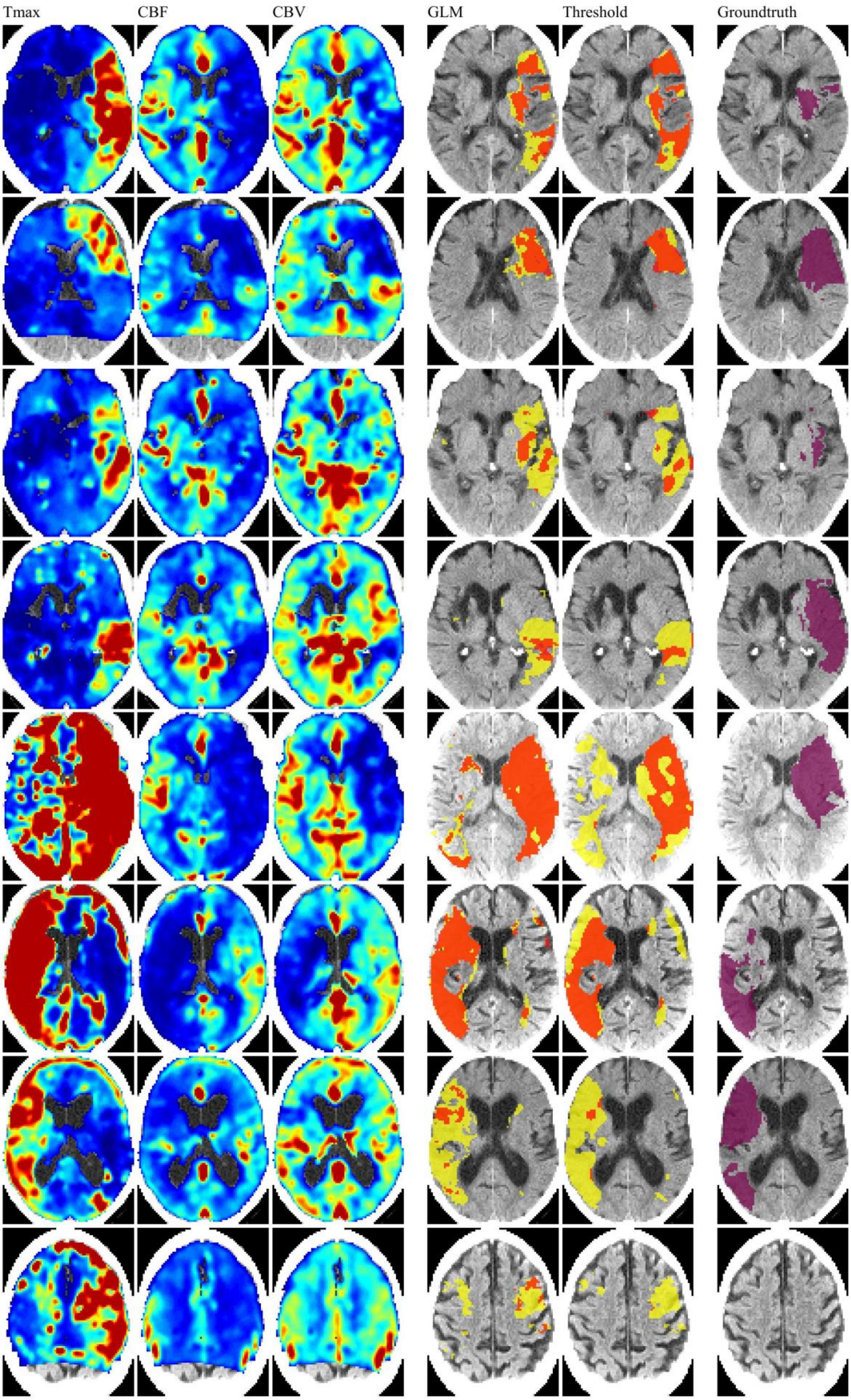

patients_id	mTICI	absVolGT	absVolDiffGLM	absVolDiffThr	diceGLM	diceThr
2345	2b	40ml	78.5ml	92.2ml	35%	29%
2334	3	125ml	81.4ml	69.8ml	51%	56%
2314	0	26.1ml	96.2ml	68.2ml	32%	34%
2321	2b	129.4ml	104.7ml	117.2ml	28%	8%
2330	3	184.5ml	105.3ml	43.9ml	70%	63%
2336	3	167.6ml	109.8ml	53.4ml	69%	66%
2313	2b	148.8ml	110.4ml	125.5ml	36%	14%
2343	3	0ml	120.4ml	53.3ml	ø	ø

Individual Predictions (Leipzig test cohort)

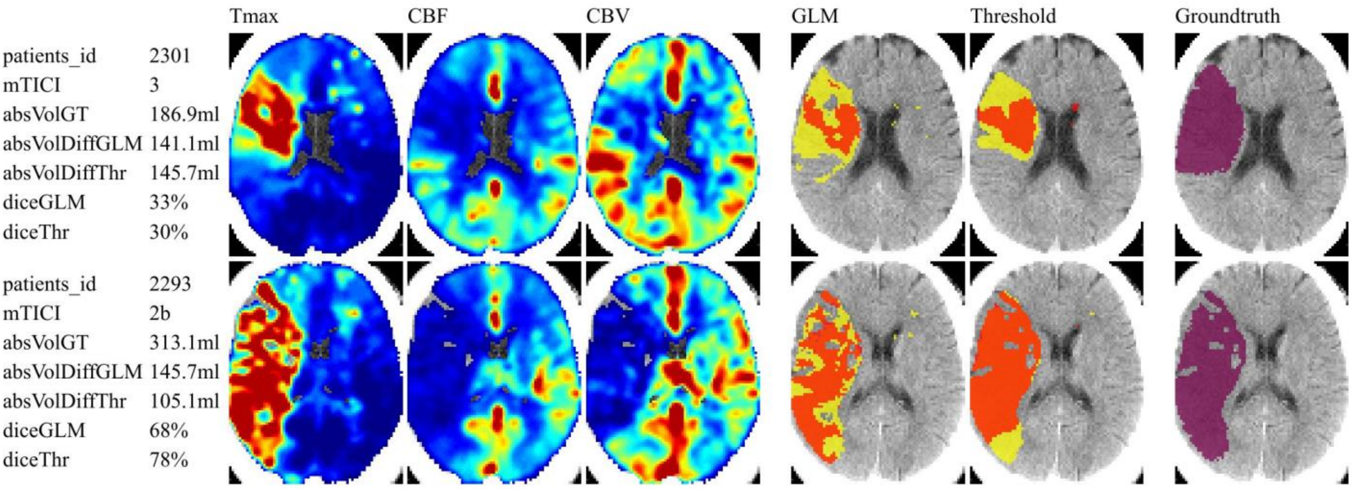

Supplement: Supplementary file 1 — Supplementary file1 (PDF 4597 KB) [file 12975_2023_1160_MOESM1_ESM.pdf]
